# Do Autistic Traits Correlate with Post-Traumatic Stress and Mood Spectrum Symptoms among Workers Complaining of Occupational Stress?

**DOI:** 10.3390/ijerph19073809

**Published:** 2022-03-23

**Authors:** Liliana Dell’Osso, Rodolfo Buselli, Martina Corsi, Sigrid Baldanzi, Carlo Antonio Bertelloni, Riccardo Marino, Davide Gravina, Martina Chiumiento, Antonello Veltri, Gabriele Massimetti, Fabrizio Caldi, Salvio Perretta, Rudy Foddis, Giovanni Guglielmi, Alfonso Cristaudo, Claudia Carmassi

**Affiliations:** 1 Psychiatric Clinic, Department of Clinical and Experimental Medicine, University of Pisa, Via Roma 67, 56100 Pisa, Italy; liliana.dellosso@med.unipi.it (L.D.); carlo.ab@hotmail.it (C.A.B.); davide.gravina@hotmail.it (D.G.); gabriele.massimetti@med.unipi.it (G.M.); ccarmassi@gmail.com (C.C.); 2 Occupational Health Department, Azienda Ospedaliero Universitaria Pisana, Via Paradisa 2, 56124 Pisa, Italy; r.buselli@gmail.com (R.B.); sigridbaldanzi@gmail.com (S.B.); riccardomarino145@gmail.com (R.M.); martina.chiumiento@gmail.com (M.C.); antonelloveltri@gmail.com (A.V.); f.caldi@ao-pisa.toscana.it (F.C.); salvio.perretta@gmail.com (S.P.); rudy.foddis@med.unipi.it (R.F.); g.guglielmi@ao-pisa.toscana.it (G.G.); a.cristaudo@gmail.com (A.C.)

**Keywords:** autistic traits, autism spectrum disorder, mood spectrum, post-traumatic stress spectrum, occupational stress, rumination

## Abstract

The adult autism subthreshold spectrum model appears to be a useful tool for detecting possible vulnerability factors in order to develop mental disorders in the contest of work-related stress. The aim of the present study is to analyze the relationship between autism, mood, and post-traumatic spectrum in a cohort of subjects complaining of work-related stress before the COVID-19 pandemic. The authors carried out a retrospective investigation of both medical records and self-assessment tools of a sample of subjects evaluated at the Occupational Health Department of a University hospital in central Italy. Data showed significant correlations between the AdAS spectrum, TALS-SR, and MOODS total and domain scores. A multiple linear regression evidenced that both the AdAS spectrum and TAL-SR significantly predict the MOODS scores. In particular, mediation analysis showed both a direct and indirect, mediated by TALS-SR, effect of the AdAS Spectrum on the MOODS-SR. These results corroborate the role of autistic traits in influencing the traumatic impact of work-related stress and the development of mood spectrum symptoms.

## 1. Introduction

Occupational stress is an important health and safety burden that arises when individual resources are not adequate to cope with the demands of a work environment [1]. It is a global phenomenon, with around half of European workers considering stress to be common in their workplace. It has also been demonstrated that stress contributes to about half of all lost working days [2,3]. Exposure to regular and repeated work-related stress can also lead to occupational mental disorders [4,5]. In this regard, in recent years, the World Health Organization (WHO) has promoted the need to preserve mental health in the workplace, given the relationship between stress and significant health and economic consequences [6].

The COVID-19 pandemic has led to a significant surge in occupational stress across different categories of workers. Literature discussing this topic has already emerged, giving us the opportunity to better investigate the constructs underlying this phenomenon, particularly among the most vulnerable subjects [7,8]. Nevertheless, international literature on work stress, to date, has been more focused on studying psychosocial risk factors closely linked to work organization, leaving a gap in defining a profile of subjects who are most likely to encounter occupational stress and negative health outcomes [9].

There is hard evidence demonstrating that phenotypic temperamental characteristics and endophenotypic elements are responsible for vulnerability to both negative and positive environmental influences [10,11]. Specific inherited characteristics, early life experiences, and, in particular, learned cognitive predispositions make subjects more or less vulnerable to the effects of stressors and occupational impairment [12,13].

An interesting personological profile that has been studied in recent years is that of subjects with autistic traits. Autistic traits, also known as “broad autism phenotype” or “subthreshold autism”, are a set of behavioral and cognitive features of autism spectrum disorder (ASD), which have been shown to continuously distribute across the general population [14,15].

Socio-cognitive features of autistic traits, such as information perception and processing, ruminative thinking, mental rigidity, emotion regulation challenges, and difficulty establishing or maintaining goal direction, may reduce the capacity of cognitive coping and other adaptive strategies to process emotions, leading to differential trauma responses [16,17]. In addition, autistic traits may influence which events are experienced as particularly traumatic (e.g., social insults and degradation, sensory overstimulation, modifications in everyday routines) and affect both the manifestation and severity of mental sequelae [18,19]. The difference in cognitive flexibility and difficulty to shift focus may, in fact, lead to increased rumination, which seems to be a main element of the autistic spectrum and one that contributes to rendering subjects prone to the perpetration of traumatization through their life [20]. For this reason, we would like to more accurately investigate the relationship between autistic traits and traumatic events such as occupational stress.

The core characteristics of adults with autistic traits are challenges in social communication and social interaction [21]. They may struggle with social skills and understanding social cues leading to negative performance reviews, demotion, or loss of employment. Further, subjects can be easily misinterpreted and may be poorly tolerated in a competitive workplace [22,23]. Some studies evidenced how subjects with autistic traits present lower occupational self-efficacy and higher rates of mental health issues than those with lower levels of autistic traits. These individuals are also more likely to desire employment-based support than individuals with lower levels of autistic traits, indicating that this may be a population that would benefit from sustained support [24].

There is also significant evidence about the possible role of autistic traits in the development of different mental disorders [25]. Some evidence points to mood and post-traumatic disorders, even suicidal thoughts and behaviors, being common in individuals with autistic traits [20,26,27]. Studies in non-clinical samples highlighted that autistic traits were positively associated with a higher incidence of bullying, with a history of physical and sexual abuse, and even with symptoms of anxiety, depression, and post-traumatic stress [28]. In particular, some authors reported elevated autistic traits levels among female nurses with a history of post-traumatic stress symptoms following childhood abuse [29,30,31]. Other associations were found between autistic traits and fibromyalgia, in particular among females [32]. Furthermore, a correlation between autistic traits and post-traumatic stress symptoms was also reported in caregivers of children with serious illnesses [33].

The autism spectrum model seems to be a useful tool for the authors’ purpose of studying vulnerability factors for developing mental symptoms in the contest of work-related stress.

The present cross-sectional investigation fits into this context with the aim of better understanding the relationship between autism, mood, and post-traumatic spectrum symptoms in a cohort of subjects complaining of work-related stress before the COVID-19 pandemic.

The authors hypothesized that those with higher levels of autistic traits were more likely to have post-traumatic stress and depressive symptoms.

## 2. Materials and Methods

### 2.1. Study Design and Setting

The authors carried out a cross-sectional collection of data by means of a retrospective investigation of both medical records and self-assessment tools of a sample of subjects evaluated at the Occupational Health Department of a University hospital in central Italy. The data collection period was from 2016 to 2018 inclusive. The pre-COVID-19 period was chosen to avoid the risk of bias due to the particular work situation that resulted from the pandemic in Italy (such as precarious work, lay-offs, and smart working).

The Occupational Health Department included a multidisciplinary group constituted by occupational physicians, psychiatrists, and psychologists. Both clinical and self-report questionnaires, based on the transactional model of stress and the workers’ subjective perception of psychosocial risks, were used in order to assess the degree of occupational stress and the possible diagnosis of occupational disease: Post-Traumatic Stress Disorder (PTSD)/Adjustment Disorder (AD).

The study was conducted in accordance with the Helsinki Declaration, and study participants signed informed consent to data acquisition.

### 2.2. Participant Recruitment: Study Inclusion and Exclusion Criteria

All those attending the outpatient clinic in the chosen period and complaining about subjective occupational stress were included in the study. Subjects with inadequate comprehension of Italian that interfered with the completion of self-report questionnaires were excluded from the study.

### 2.3. Data Sources, Instruments and Tools

Collected data consisted of demographic and work variables, lifetime mental health elements, and spectrum tools. These last are set instruments (namely, the Adult Autism Subthreshold Spectrum (AdAS Spectrum), the Mood Spectrum Self Report (MOODS-SR), and the Trauma and Loss Spectrum Self Report (TALS-SR)) developed and validated in the framework of the international research network called Spectrum Project [34]. The spectrum instruments are not intended to be used as diagnostic tools but rather to provide a dimensional assessment of psychopathology, including prodromal, residual, and subthreshold conditions.

Data from the AdAS, TALS-SR, and MOODS-SR were collected and analyzed by professionals who were trained and certified in the use of the tools.

### 2.4. Lifetime Spectrum Questionnaires

The AdAS Spectrum questionnaire has been developed to detect the presence of a wide range of ASD symptoms in adults with average intelligence and without language impairment. It allows the evaluation of a broad array of features, from threshold level ASD to partial and subthreshold forms, down to isolated autistic traits. It is composed of 160 dichotomous items, grouped in seven domains that investigate (1) the symptoms present in childhood or adolescence, (2) verbal communication, (3) non-verbal communication, (4) empathy, (5) the routine habits and inflexibility, (6) narrow interests and ruminations, and (7) hypo/hyper-responsiveness to stimuli. In the validation study, the questionnaire showed excellent reliability, with a Kuder–Richardson’s coefficient of 0.964. Moreover, the AdAS Spectrum demonstrated a high correlation with widely used measures of autism, such as the Autism-Spectrum Quotient (Pearson’s r correlation = 0.77) and the Ritvo Autism and Asperger Diagnostic Scale 14-item version (Pearson’s r correlation 0.83) [35].

The TALS-SR questionnaire is comprised of 116 dichotomous items (grouped in nine domains), assessing stress response symptoms across 3 different dimensions: (1) the dimension of loss events and potentially traumatic events, even of mild/moderate severity; (2) the dimension of the peritraumatic and acute reactions; and (3) the dimension of the post-traumatic symptoms. Using a spectrum approach, the questionnaire has the objective to investigate not only full-blown trauma and stressor-related disorders but also the broad dimension of symptoms that may follow stressful experiences across the lifetime. In the validation paper, all Kuder–Richardson coefficients for TALS-SR exceeded the minimum standard of 0.50, and the instrument demonstrated positive correlations between domains, with Pearson’s r ranging from 0.46 to 0.76 [36,37].

The MOODS-SR is a 161-item questionnaire that assesses a broad spectrum of mood symptoms, from symptom criteria and clinical level mood disorders, to mild manifestations as expressions of subclinical, prodromic, residual, and atypical syndromes [38]. Items are grouped into three manic/hypomanic and three depressive domains (exploring, in each case, the areas of mood, energy, and cognition). A further domain explores disturbances in rhythmicity and vegetative functions. The questionnaire showed good internal consistency, with a Kuder–Richardson’s coefficient ranging from 0.79 to 0.92 among single domains.

The MOODS-SR includes 140 items exploring dimensional symptoms, coded as present or absent for one or more periods of at least 3–5 days throughout the subject’s lifetime. The tool is organized into manic and depressive components (each one sub-typed into three domains exploring mood, energy, and cognition), and it also includes a section that assesses alterations in rhythmicity and vegetative functions, yielding a total of seven domains. (further information and PDF of the tool on www.spectrum-project.org (accessed on 22 March 2020)).

The internal consistency between the self-report (MOODS-SR) and the interview format (SCI-MOODS) of the MOODS always exceeded the threshold of 0.88, defining substantial reliability in all domains [38].

### 2.5. Statistical Analyses

In descriptive statistics of categoric variables, the authors determined the absolute and relative frequencies (n, %), whereas with quantitative continuous variables, we applied mean scores and standard deviations (mean ± SD) were specified.

Bivariate Pearson’s correlation coefficients were computed to investigate possible associations between the AdAS Spectrum domains and those of TALS-SR and MOODS-SR.

A hierarchical multiple linear regression model controlled by Sex was utilized to study if TALS and AdAS total scores can be considered good predictors of the MOOD-SR total score (dependent variable). Zero-order and partial correlation were also calculated to better determine which was the best predictor. Both the TALS and AdAS total scores resulted significantly associated with the dependent variable, with the second predictor slightly more correlated. Finally, a mediation analysis was performed, providing the AdAS total score as predictor, the MOOD-SR total score as dependent variable, and the TALS-SR total score as mediator. Hayes’s PROCESS tool was utilized; bootstrap confidence intervals for non-standardized and standardized indirect effect were computed.

All statistical analyses were carried out using the software package “Statistical Package for Social Science” (SPSS), version 25.0. All statistical analyses were carried out using the software package “Statistical Package for Social Science” (SPSS), version 25.0.

## 3. Results

### 3.1. Sample Characteristics and Tools Scores

A pool of different types of workers was tested and examined at the Occupational Health Department of a major University Hospital in Italy. A total of 345 subjects was screened, 188 of which were women (54.7%) and 156 men (45.3%), with a mean age of 49.8 ± 8.7 years. Other characteristics of the overall sample are set out in Table 1.

Within the whole sample, AdAS Spectrum, TALS-SR, and MOODS total mean scores (±SD) were 34.10 (±21.12), 37.15 (±18.52), and 47.26 (±25.09), respectively. See Table 2 for the other domains’ endorsement scores.

### 3.2. Correlating Analysis

Correlating the AdAS Spectrum and the MOODS-SR domains scores, good correlations emerged between the AdAS spectrum total scores and the depressive components of the MOODS-SR (depressive mood and depressive cognition, with r = 0.526 and r = 0.523, respectively). Other strong correlations emerged between the MOODS total scores and the AdAS spectrum domains VI (ruminations, r = 0.603) and AdAS spectrum total scores (r = 0.621). See Table 3.

Correlating the AdAS Spectrum and the TALS-SR domain scores, we found some moderate to strong correlations (see Table 4), in particular, emerged good correlations between TALS-SR total score and AdAS Spectrum domain III (non-verbal communication, r = 0.533), domain VI (ruminations, r = 0.542), domain VII (reactivity to stimuli, r = 0.510), and AdAS spectrum total score (r = 0.579).

### 3.3. Multivariate Analysis

A hierarchical multiple linear regression was performed with the MOODS-SR total scores as a dependent variable and inserting sex in the first block and the AdAS Spectrum and TALS-SR total scores in the second block of independent variables. Both the second block independent variables were identified to significantly predict the level of MOODS-SR scores, with the AdAS Spectrum showing the highest standardized coefficient (β = 0.40) and partial correlation (0.41). See Table 5.

**Figure 1:**
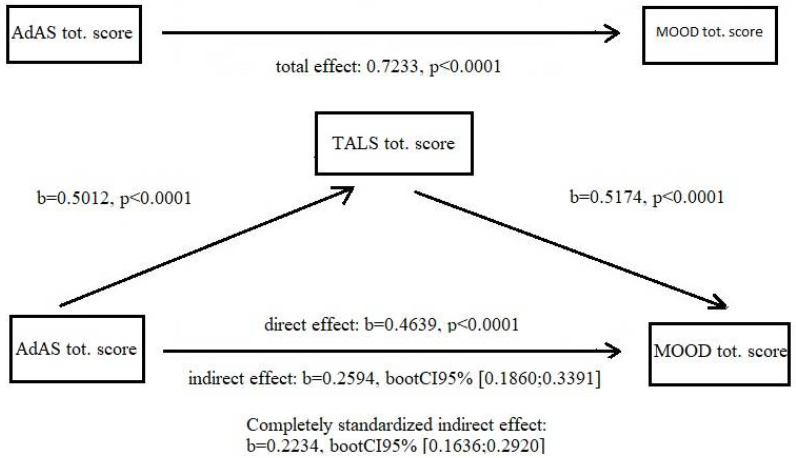
Mediation analysis results.

The mediation analysis (see Figure 1) showed that both total and direct effects of the AdAS Spectrum total score on the MOODS-SR total score were statistically significant (total effect: b = 0.7233, *p* < 0.0001; direct effect: b = 0.4639, *p* < 0.0001). The AdAS Spectrum total score also showed a significant indirect effect on the MOODS-SR total score (b = 0.2594), 95% bootstrapped CI [0.1860, 0.3391].

## 4. Discussion

To the best of our knowledge, this is the first study that aims at analyzing the relationship between autistic traits, post-traumatic stress, and mood symptoms in a population of workers complaining of occupational stress and how autistic traits interact with trauma/stressor-related symptomatology and mood symptoms. We analyzed psychopathological spectrum elements distributed over a continuum lifetime rather than personality traits, which we believe has not been done in prior studies.

In this regard, we found that certain subdomains of the autism spectrum, in particular non-verbal communication and ruminative thinking, present strong correlations with TALS-SR total scores and with the depressive components of MOODS, as well as MOODS total scores.

Non-verbal communication is considered, in fact, the strongest form of communication between coworkers since more than 90% of communication is non-verbal. Not being able to see the non-verbal cues, gestures, posture, and general body language can make communication less effective, and this can lead to perceiving work situations as more stressful [39,40]. At the same time, rumination, as a negative pattern of repetitive thinking, often affects problem solving and the ability to cope with negative feelings, exacerbating mood symptoms [41,42].

Literature evidence has highlighted that ruminative thinking is linked with a number of negative physical and psychological health outcomes, including higher risk of cardiovascular disease, risk of stroke, increased cortisol secretion, depressed mood, weakness, sleep difficulties, and exhaustion. Furthermore, longitudinal data have also highlighted that work-related rumination dramatically increased exhaustion and reduced psychological well-being [43,44]. In addition, in response to stressful life events, a ruminative attitude seems to increase the risk of developing depressive episodes and is positively related to the length and severity of a depressive episode [41,42,43,44].

Consistent with these results, some of us evidenced similar findings in other groups of traumatized subjects. In particular, what emerged from previous studies is that subjects exposed to severe illness in one’s child presented strong correlations in the same subdomains of the AdAS spectrum and TALS-SR [33]. Other studies on the same issue evidenced how autistic traits in parents of autistic children seem to be associated with a higher vulnerability toward psychopathology and with a lower global functioning [45]. Another study among police officers evidenced that intrusive ruminations served as a predictor that intensifies post-traumatic stress symptoms in the context of occupational stress [45,46]. Several approaches in occupational stress research assume, in fact, that work-related rumination prolongs stress-related affective and physiological activation and hence contributes to impairing somatic health in the long run [47].

Our results are also in line with the literature on adults with ASD. A recent meta-analysis evidenced a pooled estimate of any current anxiety and depression of 27% and 23%, respectively, in people with autism spectrum with respect to 1–12% in the general population suggesting a close connection between autistic traits and psychopathological elements [48]. This paper confirms the difficulties in estimating the exact prevalence of these traits, given the high levels of heterogeneity across studies, despite increasing results suggesting they are significantly high [48].

In this regard, the multiple linear regression model showed that both autistic traits and trauma/stressor-related symptoms predict higher levels of mood symptoms. In particular, a mediation analysis suggested a direct effect of autistic traits on affective scores mediated by trauma/stressor-related symptoms. What emerged suggests that autistic traits could influence the perception of events as traumatic and then the development of symptomatic elements in the mood field.

In this regard, since the elements that have the greatest impact on post-traumatic stress symptomatology and depression are rumination and non-verbal communication, it would be interesting to conduct a deeper analysis of these constructs in different clusters of workers.

Our findings must be considered in light of some limitations. First, the sample size was relatively small, and further studies with larger samples are needed to confirm our findings. Second, the cross-sectional retrospective design of the study prevented us from better evaluating temporal relationships between the considered variables. Moreover, the use of self-reporting instruments may have led to over- or underestimations of symptoms by the subjects and to consequent biases in our results. Finally, this was an exploratory study, and we did not differentiate in our analyses between gender, age, current mood episodes, or other elements that could affect the findings limiting the extensibility of the present work. Other interesting aspects that could be deepened in future studies are the psychosocial challenges offered by COVID-19 and how these can be experienced by subjects with subthreshold or personological autistic traits in a work context.

## 5. Conclusions

Despite the above limitations, this study sheds some light on the relationship between autistic traits, lifetime post-traumatic stress symptoms, and the mood spectrum in a sample of workers complaining of occupational stress. The strength of starting to evaluate personological spectrum elements in workers is that it can help on defining tools and strategies that can be used to train employers and occupational physicians. The long-range goal is to minimize the development of mental disorders and to improve work performance.

## Figures and Tables

**Table 1:** Sociodemographic and work-related characteristics of the overall sample (N = 345) with mean ± SD age of 49.8 ± 8.7.

	N (%)
***Gender***	
***Male***	156 (45.3%)
***Female***	188 (54.7%)
***Age***	
***<50 years***	173 (50.3%)
***>50 years***	171 (49.7%)
***Education level***	
***Primary/high school diploma***	233 (67.5%)
***At least college degree***	111 (32.3%)
***Psychiatric family history***	
***Negative***	240 (69.8%)
***Positive***	104 (30.2%)
***Psychiatric comorbidities***	
***Negative***	261 (75.9%)
***Positive***	83 (24.1%)
***Physical comorbidities***	
***Negative***	264 (76.7%)
***Positive***	80 (23.3%)
***Employment type***	
***Industries/services***	252 (73.0%)
***Education***	24 (7.0%)
***Healthcare***	60 (17.4%)
***Law officers/army***	8 (2.3%)
***Employment sector***	
***Public***	122 (35.5%)
***Private***	222 (64.5%)
***Company dimension***	
***Small (<100 p)***	101 (29.4%)
***Big (>100 p)***	243 (70.6%)

**Table 2:** AdAS, TALS, and MOOD single and total domains’ mean (±SD) scores (N = 325).

AdAS Domains	(Mean ± SD)	TALS Domains	(Mean ± SD)	MOOD Domains	(Mean ± SD)
***(I) Childhood/adolescence***	3.70 ± 2.79	***(I) Loss events***	4.44 ± 2.13	***(I) Depressive mood***	8.20 ± 6.36
***(II) Verbal communication***	3.53 ± 2.88	***(II) Grief reactions***	9.05 ± 5.34	***(II) Manic mood***	8.38 ± 4.90
***(III) Non-verbal communication***	5.21 ± 3.96	***(III) Potentially traumatic events***	5.12 ± 3.08	***(III) Depressive energy***	3.18 ± 2.99
***(IV) Empathy***	2.93 ± 2.57	***(IV) Responses to traumatic event***	6.90 ± 4.18	***(IV) Manic energy***	3.87 ± 2.81
***(V) Routinariety/inflexibility***	9.94 ± 6.70	***(V) Re-experiencing***	3.31 ± 2.53	***(V) Depressive cognition***	8.13 ± 6.40
***(VI) Narrow interests/ruminations***	5.75 ± 4.09	***(VI) Avoidance and numbing***	3.62 ± 3.23	***(VI) Manic cognition***	5.73 ± 3.87
***(VII) Hypo/hyperresponsiveness to stimuli***	3.02 ± 2.80	***(VII) Maladaptive coping***	0.90 ± 1.39	***(VII) Rhythmicity and vegetative function***	9.77 ± 5.66
		***(VIII) Arousal***	2.05 ± 1.85	***Tot man***	17.98 ± 10.01
		***(IX) Personality/risk factors***	1.62 ± 1.39	***Tot dep***	19.51 ± 14.27
***Total***	34.10 ± 21.12	***Total***	37.15 ± 18.52	***Total***	47.26 ± 25.09

**Table 3:** Correlation between AdAS spectrum and MOODS-SR total and single domain scores in the total sample (N = 318).

MOODS-SR AdAS Spectrum	(I) Depressive Mood *r,p*	(II) Manic Mood *r,p*	(III) Depressive Energy *r,p*	(IV) Manic Energy *r,p*	(V) Depressive Cognition *r,p*	(VI) *Manic Cognition r,p*	(VII) *Rhythmicity and Vegetative Function r,p*	Total *r,p*
***(I) Childhood/adolescence***	0.400	0.374	0.297	0.393	0.357	0.371	0.359	0.487
	0.000	0.004	0.000	0.000	0.000	0.002	0.000	0.000
***(II) Verbal communication***	0.403	0.358	0.296	0.382	0.425	0.357	0.328	0.492
	0.000	0.000	0.000	0.000	0.000	0.000	0.000	0.000
***(III) Non-verbal communication***	**0.516**	0.425	0.362	0.436	0.497	0.396	0.389	**0.587**
	0.000	0.000	0.000	0.000	0.000	0.000	0.000	0.000
***(IV) Empathy***	0.241	0.133	0.110	0.186	0.244	0.108	0.159	0.243
	0.000	0.018	0.050	0.001	0.000	0.055	0.001	0.000
***(V) Routinariety/inflexibility***	0.418	0.382	0.282	0.443	0.423	0.381	0.354	**0.515**
	0.000	0.000	0.000	0.000	0.000	0.000	0.000	0.000
***(VI) Narrow interests/ruminations***	0.497	0.435	0.396	0.504	0.496	0.423	0.404	**0.603**
	0.000	0.000	0.000	0.000	0.000	0.000	0.000	0.000
***(VII) Hypo/hyperresponsiveness to stimuli***	0.479	0.379	0.351	0.353	0.492	0.355	0.297	**0.529**
	0.000	0.000	0.000	0.000	0.000	0.000	0.000	0.000
***Total***	**0.526**	0.449	0.373	0.493	**0.523**	0.434	0.417	**0.621**
	0.000	0.000	0.000	0.000	0.000	0.000	0.000	0.000

Bold: good correlations: r 0.50–0.60; strong correlations: >0.60.

**Table 4:** Correlation between AdAS spectrum and TALS-SR total and single domain scores in the total sample (N = 323).

TALS-SR AdAS Spectrum	(I) Loss Events *r,p*	(II) Grief Reactions *r,p*	(III) Potentially Traumatic Events *r,p*	(IV) Responses to Traumatic Event *r,p*	(V) Re-experiencing *r,p*	(VI) Avoidance and Numbing *r,p*	(VII) Maladaptive Coping *r,p*	(VIII) Arousal	(IX) *Personality/Risk Factors*	Total *r,p*
***(I) Childhood/adolescence***	0.363	0.504	412.000	0.291	0.242	0.326	0.348	0.225	0.391	0.489
	0.000	0.004	0.000	0.000	0.000	0.002	0.000	0.000	0.000	0.000
***(II) Verbal communication***	0.245	0.475	0.354	0.235	0.193	0.250	0.350	0.187	**0.486**	**0.429**
	0.000	0.000	0.000	0.000	0.000	0.000	0.000	0.000	0.000	0.000
***(III) Non-verbal communication***	0.364	0.537	0.430	0.311	0.287	0.367	0.361	0.239	**0.525**	**0.533**
	0.000	0.000	0.000	0.000	0.000	0.000	0.000	0.000	0.000	0.000
***(IV) Empathy***	0.154	0.254	0.177	0.117	0.083	0.167	0.174	0.058	0.268	0.226
	0.000	0.000	0.001	0.036	0.137	0.003	0.002	0.295	0.000	0.000
***(V) Routinariety/inflexibility***	0.319	0.494	0.372	0.293	0.281	0.337	0.330	0.273	0.488	0.494
	0.000	0.000	0.000	0.000	0.000	0.000	0.000	0.000	0.000	0.000
***(VI) Narrow interests/ruminations***	0.320	**0.512**	0.385	0.319	0.326	0.415	0.424	0.346	0.490	0.542
	0.000	0.000	0.000	0.000	0.000	0.000	0.000	0.000	0.000	0.000
***(VII) Hypo/hyperresponsiveness to stimuli***	0.269	0.366	0.366	0.373	0.368	0.422	0.483	0.352	0.419	**0.510**
	0.000	0.000	0.000	0.000	0.000	0.000	0.000	0.000	0.000	0.000
***Total***	0.366	0.566	0.445	0.347	0.323	0.409	0.432	**0.307**	**0.554**	**0.579**
	0.000	0.000	0.000	0.000	0.000	0.000	0.000	0.000	0.000	0.000

Bold: good correlations: r 0.50–0.60; strong correlations: >0.60.

**Table 5:** Hierarchical linear model of predictors of MOOD total score: sex as predictive variable in the first block and Tals and AdAS total scores as predictive variables in the second block.

*Step 1*	b (S.E.)	β	CI_95%_	*p*	Zero-Order Correlation	Partial Correlation
***Constant***	41.85 (4.49)	-	33.03;50.68	<0.001	-	-
***Sex***	3.30 (2.78)	0.07	−2.16;8.76	=0.236	0.07	0.07
***Step 2***	**b (S.E.)**	**β**	**CI_95%_**	***p***	**Zero-Order Correlation**	**Partial Correlation**
***Constant***	11.95 (3.67)	-	4.72;19.17	=0.001	-	-
***Sex***	0.21 (2.02)	0.01	−3.77;4.18	=0.918	0.07	0.07
***TALS total score***	0.52 (0.07)	0.39	0.38;0.65	<0.001	0.61	0.40
***AdAS total score***	0.46 (0.06)	0.40	0.35;0.58	<0.001	0.62	0.41

R2 = 0.004 for step1; ΔR2 = 0.484 for step 2. β: beta regression coefficient; b (S.E.): b standard error.

## Data Availability

Datasets are available upon request to authors.
